# Additive and Lithographic Manufacturing of Biomedical Scaffold Structures Using a Versatile Thiol-Ene Photocurable Resin

**DOI:** 10.3390/polym16050655

**Published:** 2024-02-28

**Authors:** Michael Kainz, Stjepan Perak, Gerald Stubauer, Sonja Kopp, Sebastian Kauscheder, Julia Hemetzberger, Adrián Martínez Cendrero, Andrés Díaz Lantada, Disha Tupe, Zoltan Major, Dominik Hanetseder, Veronika Hruschka, Susanne Wolbank, Darja Marolt Presen, Michael Mühlberger, Elena Guillén

**Affiliations:** 1 Functional Surfaces and Nanostructures, Profactor GmbH, 4407 Steyr-Gleink, Austria; stjepan.perak@upnano.com (S.P.); gerald.stubauer@profactor.at (G.S.); sonja.kopp@profactor.at (S.K.); michael.muehlberger@profactor.at (M.M.); 2 Department of Mechanical Engineering, Universidad Politécnica de Madrid, 28006 Madrid, Spain; adrian.mcendrero@upm.es (A.M.C.); andres.diaz@upm.es (A.D.L.); 3 Institute of Polymer Product Engineering, Johannes Kepler University, 4040 Linz, Austria; disha.tupe@jku.at (D.T.); zoltan.major@jku.at (Z.M.); 4 Ludwig Boltzmann Institute for Traumatology, The Research Centre in Cooperation with AUVA, 1200 Vienna, Austria; dominik.hanetseder@trauma.lbg.ac.at (D.H.); veronika.hruschka@trauma.lbg.ac.at (V.H.); susanne.wolbank@trauma.lbg.ac.at (S.W.); darja.marolt@trauma.lbg.ac.at (D.M.P.); 5 Austrian Cluster for Tissue Regeneration, 1200 Vienna, Austria

**Keywords:** additive manufacturing, biomimetic scaffold, photopolymerisation, thiol-ene

## Abstract

Additive and lithographic manufacturing technologies using photopolymerisation provide a powerful tool for fabricating multiscale structures, which is especially interesting for biomimetic scaffolds and biointerfaces. However, most resins are tailored to one particular fabrication technology, showing drawbacks for versatile use. Hence, we used a resin based on thiol-ene chemistry, leveraging its numerous advantages such as low oxygen inhibition, minimal shrinkage and high monomer conversion. The resin is tailored to applications in additive and lithographic technologies for future biofabrication where fast curing kinetics in the presence of oxygen are required, namely 3D inkjet printing, digital light processing and nanoimprint lithography. These technologies enable us to fabricate scaffolds over a span of six orders of magnitude with a maximum of 10 mm and a minimum of 150 nm in height, including bioinspired porous structures with controlled architecture, hole-patterned plates and micro/submicro patterned surfaces. Such versatile properties, combined with noncytotoxicity, degradability and the commercial availability of all the components render the resin as a prototyping material for tissue engineers.

## 1. Introduction

As a response to the demand for tissue engineering (TE) scaffolds, a diverse range of additive manufacturing technologies (AMTs) and methods have already been applied [1]. In this context, tissue engineers strive to mimic anatomical architectures ranging over several orders of magnitude through design aspects. These include porous scaffolds, allowing cellular invasion, as well as micro and nanopatterned surfaces fostering cellular growth or possessing antibacterial properties found in nature [2].

A commonly known approach for the fabrication of bioinspired structures is photopolymerisation [3,4] which sees widespread use in material jetting, vat polymerisation and nanoimprint lithography (NIL). These technologies, which are also among the methods for future biofabrication, offer the ability to create scaffolds and textured surfaces with fine and well-defined features in a short time [5]. The fundamental aspect in these manufacturing technologies is the conversion of a resin into a crosslinked-network that can maintain shape fidelity at a multiscale level. However, due to the particularities of each process, most resins are tailored to one specific technology. The method of 3D inkjet printing (IJP) is a noncontact layer by layer deposition process in which a resin is jetted onto a printing tray and subsequently cured. Digital light processing (DLP) is a vat polymerisation method in which the resin is placed in a vat and each layer is solidified by projecting light from beneath the resin vat. The build platform gradually moves upward as each layer is cured. Finally, NIL is a top-down lithographic technique used in nanofabrication. A stamp with micro or nanoscale features is pressed onto a substrate coated with a resin material. The mould transfers its pattern to the resin, and after curing, the desired structures are left on the substrate. This technique allows for the precise replication of patterns on a nanometre scale, making it a powerful tool for nanofabrication processes.

An obstacle to overcome on the journey to future biofabrication, lies in the development of highly reactive, low viscous, versatile biocompatible resins, processable with a variety of technologies with the potential to be used at a multiscale level. Using resins on different fabrication technologies, capable of realising feature sizes on various length scales, not only ensures the production of multiscale structures but also unlocks a wide range of possibilities in designing biomimetic scaffolding structures. The importance was recently emphasised by Skliutas et al. [6], as they formulated a bio-based acrylate resin and fabricated structures over a range of five orders of magnitude using DLP and nanolithography. In addition, Sänger et al. [7] employed photopolymerisation of acrylate-based ceramic slurries for the fabrication of hybrid multiscale structures ranging from several mm to the µm range using lithographic ceramic manufacturing and two-photon polymerisation, employing the same feedstock of ceramic slurry, but tailoring the absorption spectra of the initiator system to the emission spectra of the AMTs.

One strategy for formulating photocurable resins involves the exploitation of thiol-ene/yne chemistry, which has already gained substantial attention for biomedical applications [8] and for vat polymerisation of degradable polymers [9,10,11,12,13]. An already used class of monomers for AMTs and TE scaffolds are vinyl esters (VE) since they show suitable potential for biomedical applications due to their favourable cytotoxicity profile [14]. Resins containing VEs have been extensively used in various AMTs, including vat polymerisation [15,16,17,18,19,20,21], two-photon polymerisation [22,23,24] and in casting techniques [25,26,27], particularly for biological scaffolding structures. However, the usage of VEs as homopolymers lack reactivity compared to acrylates [28]. A commonly used strategy to overcome this limitation is to incorporate thiols as comonomers, which leads to a boost in reactivity [29,30].

The aim of this study was to apply a thiol-ene resin across three industrially significant technologies capable of processing photocurable resins for prototyping bioinspired scaffolds: namely vat polymerisation and especially by adding 3D inkjet printing and NIL to the existing portfolio of processing technologies for VEs. A comparative evaluation of selected bioinspired scaffolds revealed that structures over a span of six orders of magnitude, starting from the macroscale (<10 mm), moving into the microscale (<500 µm), and reaching the submicroscale (<150 nm), were successfully fabricated.

## 2. Materials and Methods

### 2.1. Materials

Divinyl adipate (DVA) was purchased from TCI Chemicals (Eschborn, Germany). Pentaerythritol tetra-3-mercaptopropionate (PETMP), propyl gallate and bis(2,4,6-trimethylbenzoyl) phenylphosphine oxide (BAPO) were obtained from Sigma-Aldrich (St. Louis, MO, USA). All chemicals were used as received.

### 2.2. Resin Characterisation

#### 2.2.1. Viscosity

Viscosity measurements were conducted on a MCR 302e rheometer (Anton Paar, Graz, Austria) with a double gap system setup. Dynamic viscosity was measured at 25 °C, 40 °C and 60 °C, at shear rates ranging from 1 to 1000 s^−1^.

#### 2.2.2. Thermal Stability

Thermal stability of the resin was evaluated by monitoring the change in viscosity over time. The resin was stored at 25 °C and 60 °C in a brown flask to avoid photo-induced polymerisation for 30 days and viscosity was measured every other day at storage temperature at a shear rate of 100 s^−1^.

#### 2.2.3. Density

The resin’s density was determined by weighing 1 mL at 25 °C and calculating it based on the mass-to-volume ratio, utilising a CPA225D mass balance (Sartorius, Göttingen, Germany).

#### 2.2.4. Surface Tension

To assess the static surface tension, a DSA100 drop shape analyser (Krüss, Hamburg, Germany) was used. Measurement was carried out at 25 °C. The pendant drop method was applied and the recorded drop shape was fitted to the Young-Laplace equation to calculate the surface tension.

#### 2.2.5. Monomer Conversion

Monomer conversion was monitored via Fourier-transform infrared (FTIR) spectroscopy in absorbance mode and was recorded using a Bruker Tensor 37 (Billerica, MA, USA) equipped with a MIRacle attenuated total reflectance bridge.

### 2.3. Fabrication Technologies

A total of four different fabrication technologies, which included two material jetting technologies, vat polymerisation, and NIL, were used to fabricate bioinspired structures spanning six orders of magnitude.

At the macroscale, structures were fabricated using an Objet Connex 500 3D industrial inkjet printer (Stratasys, Rehovot, Israel) equipped with the Ricoh Gen3 E1 printhead family (Ricoh, Tokyo, Japan) which contained 96 nozzles with a 30 pL drop volume. Curing was performed with a Cobra Cure FX1 UV-LED (ProPhotonix, Boston, MA, USA) (395 nm, 4 W/cm^2^). Syringe feeding was used to introduce the resin into the printing block. Additionally, DLP was performed with a Sonic Mini 4K (Phrozen, Hsinchu, Taiwan) which featured a ParaLED Matrix 2.0 (405 nm). The 3D models were sliced using Chitubox software (Version 1.9.5). The printing process began with a bottom exposure time of 25 s for the initial 6 layers. Subsequently, we implemented a transition layer approach with a count of 7 layers, gradually reducing the exposure time by 2.5 s per layer to a final exposure time of 5 s per layer. A uniform layer height of 50 µm was chosen. After printing, the uncured resin trapped within pores of the structures was removed by immersing the parts in propylene glycol mono methyl ether acetate and exposing them to an ultrasonic bath for 10 min.

For structures at the microscale, a PixDro LP50 (Süss MicroTec, Garching, Germany) equipped with a KM1024iSHE (Konica Minolta, Tokyo, Japan) industrial piezoelectric inkjet printhead with 1024 nozzles and 6 pL drop volume was used. Curing was performed with a FireEdge400 UV-LED (Phoseon, Hillsboro, OR, USA) (395 nm, 8 W/cm^2^). The printing parameters included a printing frequency of 6 kHz and a printing speed of 200 mm/s, resulting in a time to lamp of 75 ms. The printing temperature was 40 °C. The distance from the nozzle plate to the substrate was set to 1 mm.

Lastly, structures at the micro and submicroscale were fabricated through NIL, using Sylgard 184 (Dow, Midland, MI, USA) PDMS stamps with patterns of different bioinspired structures. Curing was performed using a UV-LED (395 nm, 180 mW/cm^2^, 15 s).

### 2.4. Accuracy and Surface Characterisation

The printing accuracy for inkjet-printed samples was assessed by measuring struts and/or pores at the midpoint of structures, both in the printing direction and perpendicular to it, using a VHX-5000 digital microscope (Keyence, Osaka, Japan). For DLP printed structures, the evaluation focused on struts using the same criteria. The topography of samples fabricated via NIL at the micro and submicroscale was analysed using a VK-X3000 laser scanning microscope (Keyence, Osaka, Japan) as well as atomic force microscopy (AFM) using a Bruker Dimension Edge microscope (Billerica, MA, USA) in tapping mode. Image processing and evaluation was performed using Gwyddion (Version 2.63) [31].

### 2.5. Bulk Material Characterisation

#### 2.5.1. Water Contact Angle

Water contact angle was measured using a DSA100 drop shape analyser (Krüss, Hamburg, Germany). Measurement was carried out at 25 °C.

#### 2.5.2. Degradation

The investigation of bulk material degradation involved a mass loss study. The process involved treating cured sample discs with a diameter of 12 mm and a height of 2 mm with ethyl acetate to remove residual unreacted resin components. Following this, the discs were dried for the determination of the initial mass value. Subsequently, the sample discs were immersed in NaHCO_3_/Na_2_CO_3_ buffer (pH 10) and incubated at 37.4 °C without agitation. At regular intervals, samples were retrieved, dried until a constant weight was achieved, and their weights were then documented. The acquired data was used for calculating bulk degradation.

#### 2.5.3. Biological Evaluation

Sample preparation: residual unreacted components were eluted using a multistep washing procedure. Therefore, cured sample discs with a diameter of 12 mm and a height of 2 mm were washed with 75% ethanol for 4 × 24 h at 37 °C under constant agitation and evacuated for an additional 24 h at 80 °C to ensure the removal of trapped solvent. Following the initial wash, the samples were washed 4 × 24 h in growth medium at 37 °C under constant agitation.

Cell culture: human primary bone marrow stromal cells (BMSCs, Lonza, Basel, Switzerland) were expanded in a growth medium consisting of Dulbecco’s modified Eagle’s medium high glucose, 10% foetal bovine serum, 100 U/mL penicillin-streptomycin, 2 mM L-glutamine (all purchased from Sigma Aldrich, St. Louis, MO, USA) and 1 ng/mL basic fibroblast growth factor (PeproTech^®^, Fisher Scientific, Pittsburgh, PA, USA). BMSCs were characterised according to the consensus position statement of the International Society for Cellular Therapy [32]. Cells of passage 4 were used for cytotoxicity experiments.

Viability assay: for cytotoxicity testing of leachable components under physiological conditions, eluates of the material were prepared. Therefore, the material was placed in a well of a 24-well culture plate, completely immersed with 1 mL growth medium and incubated for 24 h in a cell culture incubator at 37 °C and 5% CO_2_. Controls were generated by incubating the growth medium without a sample. For the 100% baseline of cell viability, cells were cultivated on cell culture plastic in growth medium. As reference for cytotoxicity, a shredded latex glove was used and eluted under the same conditions. Cells were seeded 24 h prior the experiment into a 96-well plate at 3000 cells/well. After 24 h of seeding, cells were washed with phosphate-buffered saline (PBS, Lonza, Basel, Switzerland) without Ca^2+^ and Mg^2+^. Afterwards, 100 µL of the eluate was added and cells were cultivated for an additional 24 h under cell culture conditions. Then the eluate was removed, and cells were washed with PBS. Metabolic activity was determined with growth medium supplemented with 10% (*v*/*v*) CCK-8 reagent (Merck, Darmstadt, Germany). After 2 h, absorbance at 450 nm and 650 nm was measured using the POLARstar Omega microplate reader (BMG LABTECH, Ortenberg, Germany). The absorbance difference (450 nm–650 nm) corrected by a blank was related to the controls and presented as percentage viability.

Live/dead fluorescence staining: to visualise the morphology of living cells adhering to the material, cells were seeded at a density of 20,000 cells/scaffold on the material and cultivated for 72 h. After the cultivation period, cells were washed once in PBS and then incubated in a Calcein AM (5 µM)/PI (1.5 µM) staining solution for 15 min in a cell culture incubator at 37 °C and 5% CO_2_ and protected from light. Afterwards, samples were analysed with an Axio Observer A1 microscope fitted with ICm1 AxioCam (Carl Zeiss Microscopy, Oberkochen, Germany). Images were processed using ImageJ software (Version 1.53f51).

#### 2.5.4. Mechanical Evaluation

Dynamic mechanical analysis (DMA) was performed under uniaxial loading, at a temperature range from −80 °C to +80 °C and at frequencies from 0.5 Hz to 50 Hz with the ISO 6721-4 [33] test standard geometry. The measurement was performed with an Eplexor 500N (Netzsch-Gerätebau GmbH, Selb, Germany), which started at the lowest temperature and was increased in steps of 5 K. Tensile tests were performed on an MTS servo hydraulic material testing system (MTS Systems Corporation, Eden Prairie, MN, USA) with a 250 N force cell at 25 °C and the ISO 527-2 [34] Type 5B test standard. The specimens were measured at room temperature, with loading rates of 0.1 mm/s until the specimen broke. Compression tests were tested at 0.1 mm/s loading rate and a 10 kN load cell at 25 °C using the ISO 604 [35] test standard geometry. Force and displacement data were recorded for the creation of the stress-strain curve. PDMS moulds were used to cast the specimens for the DMA, tensile and compression tests.

## 3. Results and Discussion

### 3.1. Resin Properties

Employing a resin across various manufacturing technologies necessitates meeting the distinct requirements of each method. The most relevant properties to consider are viscosity, thermal stability, curing kinetics and low oxygen inhibition. Out of all the targeted fabrication methods, inkjet printing is the most demanding technology with respect to fluid properties, which only works in a narrow viscosity window of 8–20 mPa·s between 40–60 °C and with a preferred surface tension of 28–35 mN/m. Additionally, due to high shear rates in the nozzle, reaching values up to 10^5^ s^−1^, Newtonian behaviour is desired.

We prepared a resin based on thiol-ene chemistry using a stoichiometric mixture of thiol and ene functional groups. Monomers were selected based on their suitable rheological properties ideal for inkjet printing. An additional criterion was low cytotoxicity and promising applications for biomedical purposes. Therefore, DVA, a widely recognised vinyl ester used in 3D printing [16] and PETMP, also employed for 3D printing as a comonomer [9] were used. An obstacle in the formulation of thiol-ene resins is their poor shelf and pot life, since they are known to suffer from premature gelation at elevated temperatures. The pioneering work of Liskas group laid the foundation for stabilisation of thiol-ene mixtures where pyrogallol was found to be most efficient [36]. In contrast to pyrogallol, we used propyl gallate as an alternative polyphenolic compound and additive used in the food industry known for its scavenging potential of free radicals [37] as the inhibitor at 0.5 wt.%. Finally, BAPO was chosen as the initiator as it is already used for dental materials [38] and TE scaffolds [16]. Its absorption tailing out in the visible range makes it suitable with all AMT employed and was added at a final concentration of 1 wt.%.

Viscosity measurements were conducted at three distinct temperatures, resembling temperature windows for NIL, which is commonly performed at room temperature, and DLP as well as 3D inkjet printing, operating between 25 °C and 65 °C. Viscosity measurements revealed 22 mPa·s at 25 °C, which fell within the optimal range for NIL [39] and was suitable for DLP [40]. Additionally, measurements at 40 °C yielded a viscosity of 12 mPa·s, while at 60 °C, it decreased to 6.81 mPa·s, both of which aligned with the requirements for 3D piezoelectric inkjet technologies. In addition, surface tension measurements confirmed that the resin was in the suitable range for commonly used industrial inkjet printheads. Table 1 summarises the resin properties.

The efficiency of propyl gallate as a radical scavenger to inhibit thermal polymerisation was assessed via viscosity measurements and the results are presented in Table 2. Thermal stability tests revealed a change of 3.14% over a storage period of 30 days at 25 °C as well as 27% when stored at 60 °C ensuring sufficient stability with respect to change in viscosity to be used in all fabrication technologies for a period of one month.

### 3.2. Fabrication of Bioinspired Structures

#### 3.2.1. Bioinspired Structures

**Figure 1:**
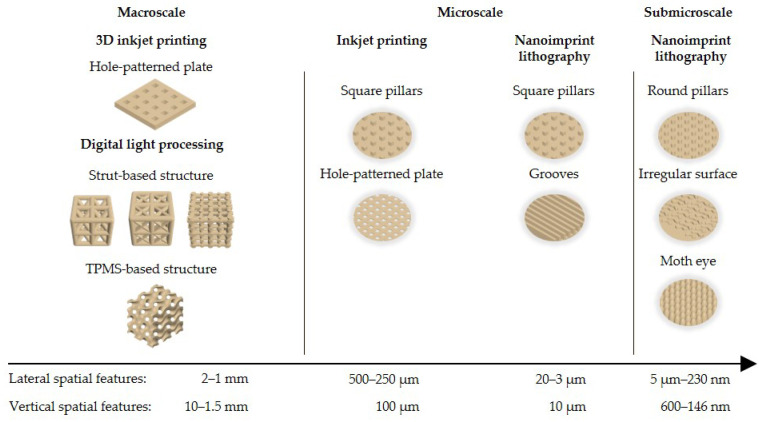
Bioinspired structures displayed according to the fabrication technologies with descending order of magnitude.

Figure 1 provides a condensed overview of the bioinspired scaffolding structures fabricated over a span of six orders of magnitude, ranging from the macro to the submicroscale. To achieve this diversity, we drew inspiration from various sources and fields of TE scaffolds. Given the complexity of interactions between cells and substrates, a universal design or size was not applicable [41]. The utility of a versatile resin became evident as it addressed diverse geometries and scales. This adaptability was advantageous for systematically identifying optimal designs tailored for specific target TE applications.

Macroscale models were among others obtained from an open-source library of established TE scaffolds considering specific features of most AMTs and containing a comprehensive collection of TE scaffold blueprints, developed by Martínez Cendrero et al. [42] and Lantada et al. [43]. Several porous models were selected that differed in unit cell types of strut-based and triply periodic minimal surface (TPMS) lattices. To demonstrate the use of the resin for the fabrication of microscale structures, nature-inspired pillar structures and hole-patterned plates were selected. For micro and submicroscale structures we presented the fabrication of different surface textured topographies. These structures, also inspired by an open scaffold library by Lantada et al. [44], which are typically used for textured geometries for studying cell-material interaction, include grooves, pillars, randomly arranged irregular topographies and a moth eye array.

#### 3.2.2. Macroscale Structures Fabricated via 3D Inkjet Printing Using PolyJet^TM^ Technology

3D inkjet printing using PolyJet^TM^ technology stands out for its exceptional resolution and its multimaterial capabilities, allowing for the use of both rigid and soft materials in a single print with minimal design limitations. PolyJet^TM^ technology has been already utilised for 3D anatomical tissue mimicking [45] and for the fabrication of scaffolding structures [46].

There are only a handful of studies that attempted to develop an ink which is compatible with PolyJet^TM^ process, including a radiopaque resin for anatomical phantoms which uses acrylate-based MED610^TM^ from Stratasys as a base material [47] and a preliminary approach for polyimide-like inks with maleimide functionalities [48]. In the current work, we reported a one component thiol-ene resin with sufficient thermal stability printed on a Connex platform, which was an important step for PolyJet^TM^ technology.

**Figure 2:**
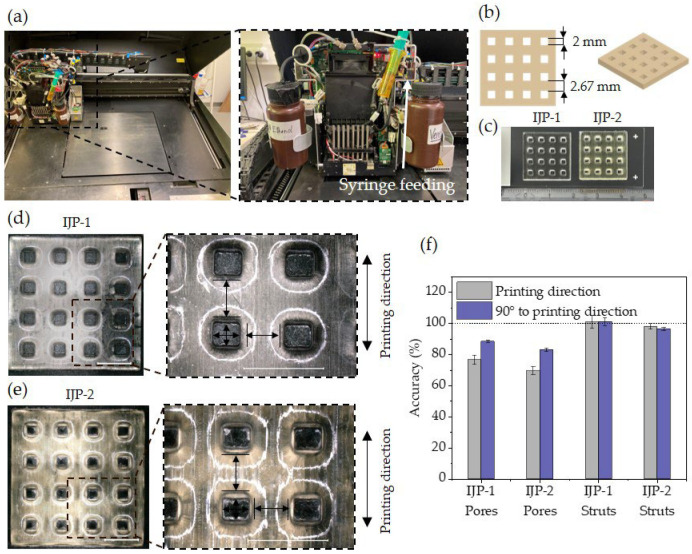
Bioinspired macroscale structures fabricated via 3D inkjet printing using PolyJet^TM^ technology: (**a**) Objet 500 Connex platform. Close up view showing the printing block with syringe feeding; (**b**) hole-patterned plate design; (**c**) photograph of the two printed hole-patterned plates; (**d**,**e**) top view of the structure imaged via optical microscopy. Close up view with indicated measurement positions. Scale bar: 5 mm; (**f**) accuracy measurement of pores and struts normalised to the design.

A noteworthy aspect of our work is that we are uniquely equipped to have an open access Connex platform to study our resin. Syringe feeding, as presented in Figure 2a, was employed, allowing for the direct supply of material to the printheads. This method enabled the evaluation of experimental materials in small quantities, thereby exploring new possibilities for testing inks using PolyJet^TM^ technology. Two quadratic mono-material hole-patterned plate models with a side length of 20 × 20 mm^2^ were printed, each with a pore size of 2 mm and strut size of 2.67 mm (Figure 2b). The models, intended as a prestage for lattice structures, differed in height (h): model IJP-1 with $h_{IJP-1}$ = 1.5 mm and model IJP-2 with $h_{IJP-2}$ = 4 mm. Due to the challenge of printing overhanging structures without support material with this technology, we focused on these models to demonstrate printability.

Structures can be reliably printed and easily removed from the printing tray. Notably, the models appeared transparent in the case of thinner structures, while the thicker one exhibited yellowing due to the presence of the initiator, see Figure 2c. Optical microscopy images of the two samples, are presented in Figure 2d,e. To determine accuracy, the strut and pore size was measured, as indicated in the close-up view. The printed samples deviated slightly from the design, displaying rounded corners of the pores instead of sharp edges. As a result, the measurement position for the struts was chosen at the inflection point of the pores and measured on the top surface to assess the structural features. Pore size was measured at the bottom surface. Strut sizes matched the intended design, while pore sizes were smaller than designed for both structures, with a more pronounced deviation in the printing direction. The mismatch was particularly higher in the case of the taller structure, as illustrated in the graph in Figure 2f.

#### 3.2.3. Macroscale Structures Fabricated via Digital Light Processing

In addition to 3D inkjet printing, DLP was performed as an accessible prototyping method found in most research labs. DLP, as a vat polymerisation method, exhibits several advantages for the fabrication of lattice structures such as high speed, low cost, ease of material handling and the possibility of fabricating complex features.

**Figure 3:**
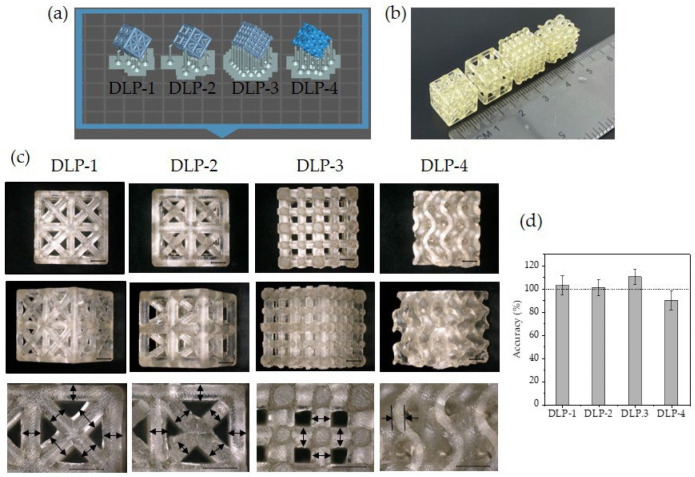
Bioinspired macroscale structures fabricated via DLP: (**a**) models imported in Chitubox software, Version 1.9.5, including support structures. Nominal dimensions 10 × 10 × 10 mm^3^; (**b**) photograph of printed structures; (**c**) structures imaged via optical microscopy for face-centred structure (DLP-1). Body-centred structure (DLP-2). Fractal cubic simple structure (DLP-3). TPMS-based structure (DLP-4). Front view (top), angled view (middle) and close-up view with indicated measurement positions (bottom). Scale bar: 2 mm; (**d**) accuracy measurement of struts normalised to the design.

Lattice structures, which are porous architectures composed of periodic arrangement of unit cells, are excellent candidates for scaffold fabrication [49]. Four models with pore and strut sizes ranging from 1 mm to 1.5 mm were selected, including strut-based structures like face-centred (DLP-1), body-centred (DLP-2), fractal cubic simple (DLP-3) and triply periodic minimal surface (TPMS) (DLP-4) lattices, see Figure 3. To print internal support-free structures, each part was tilted by 45° in both the X and Y directions, Figure 3a. Selected models were successfully printed (Figure 3b,c) using the resin. To determine accuracy of the printing, strut dimensions of the different lattices were measured and compared with designs, Figure 3d. Face- and body-centred lattices exhibited slight deviations of 3% and 1% with respect to the design. Cubic simple and gyroid lattices also displayed minor deviations from the intended design, with a measured 10% mismatch in strut size.

#### 3.2.4. Microscale Structures Fabricated via Inkjet Printing

The fabrication of structures at the microscale was investigated using another inkjet technology, namely inkjet printing on a PixDro LP50 equipped with a Konica Minolta KM1024iSHE industrial piezoelectric printhead. In contrast to PolyJet^TM^, this method employs a lower drop volume printhead, especially suited for microscale structures.

**Figure 4:**
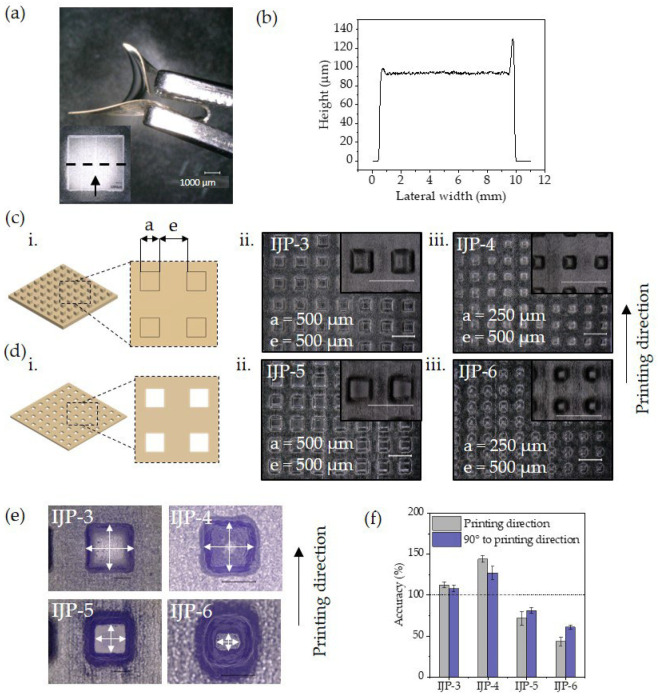
Bioinspired microscale structures fabricated via inkjet printing using a PixDro LP50 equipped with a Konica Minolta KM1024iSHE industrial piezoelectric printhead: (**a**) 10 × 10 mm^2^ printed square and qualitative image illustrating the soft polymer network. Inset showing the location of the position of the layer height measurement and the arrow indicating the printing direction; (**b**) layer height measurement of five layers; (**c**) (**i**) parametric design and (**ii**,**iii**) digital microscopic images of pillar structures; (**d**) (**i**) parametric design and (**ii**,**iii**) digital microscopic images of hole-patterned plates. Insets: View from 10° tilt. All samples printed with five layers. Scale bar: 1000 µm. Inset scale bar: 1000 µm; (**e**) Close up top view of printed structures with indication of measurement position. Scale bar: 200 µm; (**f**) accuracy measurement of pores normalised to the design.

The flexibility of the polymer network is qualitatively displayed in Figure 4a via a printed square (10 × 10 mm^2^) with five layers and a total height of 100 µm as depicted in Figure 4b. The fabrication of microstructures is presented with micropillars (Figure 4c) and hole patterned plates with a square cross-sectional shape (Figure 4d). Topographies at the submillimetre scale, such as pillars similar in size to tissue subunits, might influence the collective behaviour of cells within a group [50]. In addition, hole-patterned plates relevant for guided tissue regeneration (GTR) membranes with suitable pore size are presented. GTR membranes serve as barriers, selectively allowing the growth of desired cells while preventing the migration of undesirable cells into the healing site; they are commonly employed in periodontal surgeries to facilitate tissue regeneration. Tayebi et al. [51] suggested that pore sizes between 50–400 µm and with a thickness of 150 µm of this type of membrane can be optimal for providing the desired barrier function.

A parametric design was chosen to systematically vary the size of structures using four different patterns with feature sizes ranging from 500 µm to 250 µm side length (*a*) and a spacing between structures (*e*) of 500 µm. All structures were printed with five layers, as the square printed in Figure 4a,b.

Micropillars were successfully printed with two different side lengths (a), IJP-3 (a = 500 µm) and IJP-4 (a = 250 µm) and are depicted in Figure 4c (ii,iii). Accuracy analysis was performed by comparing the side length (*a*) with the designs, as presented in the graph in Figure 4f. The measurement position is indicated in Figure 4e. A loss of fidelity was found and as expected, this deviation was more dominant for smaller pillars. A more pronounced mismatch occurred in the printing direction with a deviation of 12% for 500 µm square pillars (IJP-3) and 44% for 250 µm square pillars (IJP-4). Perpendicular to the printing direction, the deviations were 8% for 500 µm pillars and 27% for 250 µm pillars.

Two membrane structures with pore sizes of 500 µm (IJP-5) and 250 µm (IJP-6) and a height of 100 µm were fabricated, Figure 4d (ii,iii). A comparison of the side length (*a*) of the printed holes with the design reveals that the mismatch was more dominant in printing direction, with a deviation of 28% for 500 µm pores and 57% for 250 µm pores. Conversely, deviations were 19% for 500 µm pores and 39% for 250 µm pores perpendicular to the printing direction. The loss of the square shape became noticeable as the pore size decreased, leading to a pronounced rounding in the pores with a size of 250 µm (Figure 4e).

#### 3.2.5. Micro and Submicroscale Structures Fabricated via Nanoimprint Lithography

**Figure 5:**
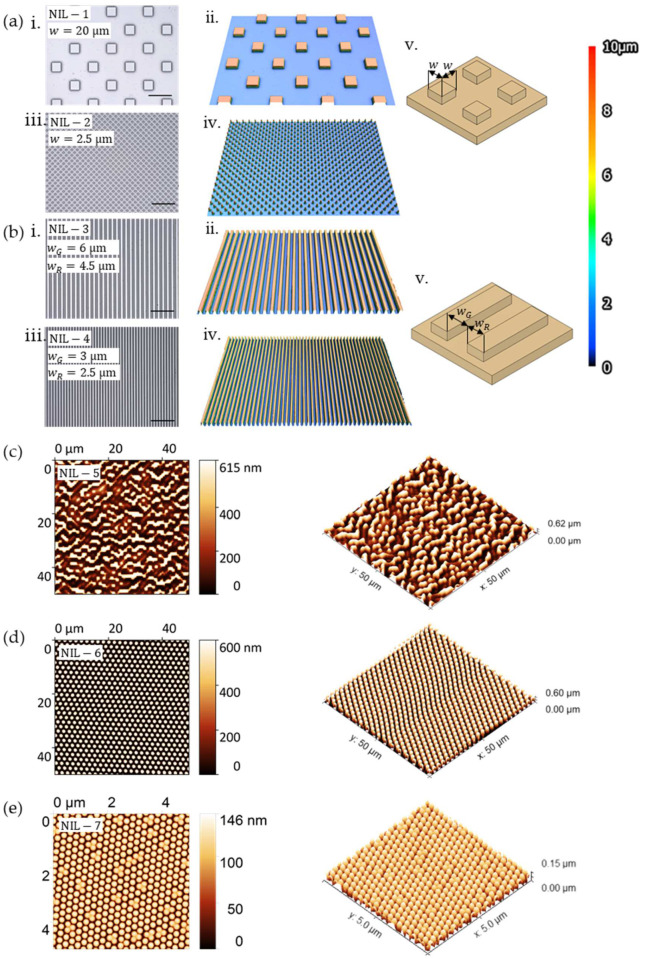
Bioinspired micro and submicroscale structures fabricated via NIL. Topography imaged via laser scanning microscopy for arrays of: (**a**) (**i**,**ii**) square pillars (NIL-1). (**iii**,**iv**) Square pillars (NIL-2). (**v**) Design dimensions for pillars; (**b**) (**i**,**ii**) Grooves (NIL-3). (**iii**,**iv**) Grooves (NIL-4). (**v**) Design dimensions for grooves. Top view scale bar: 50 µm. Colour scale applies to all images; Top and tilted (45°) topographic AFM images for arrays of: (**c**) irregular surfaces; (**d**) circular pillars; (**e**) moth eye.

The advantage of thiol-ene chemistry, respectably reduced oxygen inhibition, are especially favourable properties for NIL and the fabrication of topographical features at the micro and submicroscale. Such topographical features may influence cell behaviour encompassing adhesion, proliferation and differentiation [52,53,54]. We present the fabrication of seven different textured structures laterally in the range from 20 µm to 240 nm and from 10 µm to 150 nm with respect to height, as depicted in Figure 5. These structures included pillars, grooves and randomly arranged irregular submicroscale topographies, all designed to favour cell adhesion. Additionally, the fabrication of a moth eye array, known for its antibacterial properties, is presented.

Pillars are evenly distributed patterns on a surface and can promote cell alignment depending on the interpillar spacing, stiffness of the substrate material as well as the aspect ratio [55]. In the current work, squared micropillars with a height of 10 µm and two different side lengths, w = 20 µm (NIL-1) and w = 2.5 µm (NIL-2), were fabricated, as presented in Figure 5a.

In addition, two different microgrooved structures with a groove width of $w_{G}$ = 6 µm and a ridge width of $w_{R}$ = 4.5 µm (NIL-3) as well as $w_{G}$ = 3 µm and $w_{R}$ = 2.5 µm (NIL-4) with a height of 10 µm (Figure 5b), were replicated. This type of structure, where the groove width is commonly larger or equal to the size of a single cell, can allow cell attachment and migration, as well as cell alignment following the geometrical guidance [56]. Several studies demonstrated that cells subjected to microgroove substrates were significantly more elongated and significantly more aligned on microgroove substrates surfaces with topographical cues as compared to those on smooth surfaces [57,58].

Furthermore, three different structures below 1 µm were fabricated. Several authors have reported on the importance of nanoscale cues for cell differentiation [59] and adhesion [60,61]. An irregular surface with a height of 620 nm, a width of 2 µm and a length of 5 µm (NIL-5, Figure 5c) and circular pillars with 600 nm height and a diameter of 1 µm (NIL-6, Figure 5d) were successfully replicated. Additionally, a moth eye array (NIL-7, Figure 5e) known for its antibacterial properties [62,63], with a height of 150 nm and a diameter of 200 nm, is presented.

The results show high quality imprints for all types of structures, without irregularities and stamp removal without loss of detail for submicro structures. We observed minor defects after stamp removal for the moth eye structures, which we attributed to the softness of the material.

### 3.3. Bulk Material Properties

**Figure 6:**
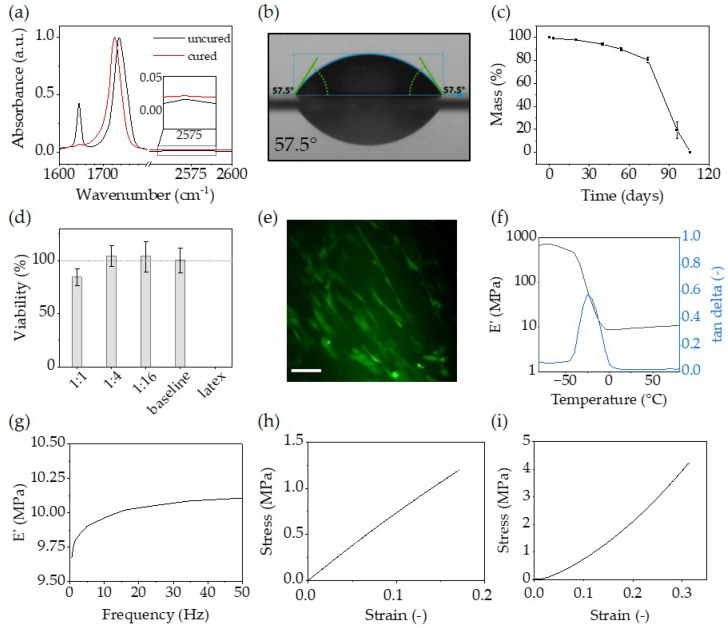
(**a**) FTIR spectrum of the uncured and cured thiol-ene resin with characteristic bands for the vinyl double bond at 1645 cm^−1^, the carbonyl bond at 1700 cm^−1^, and the S-H bond at 2570 cm^−1^; (**b**) water contact angle; (**c**) mass loss of the bulk material at 37.4 °C in percent of initial mass at pH 10; (**d**) CCK-8 viability assay of BMSCs (n = 3) with different dilutions of the material eluate, the baseline (100%) and latex eluate; (**e**) live/dead fluorescence microscopy image of viable BMSCs (green) grown for 72 h on the material surface. Scale bar: 100 µm; (**f**) storage modulus and tan delta from DMA measurement at 1 Hz; (**g**) DMA frequency sweep at 35 °C; (**h**) tensile measurement at a rate of 0.1 mm/s; (**i**) compression measurement at 0.1 mm/s.

The material properties, essential for translating the bioinspired structures presented into possible use cases, are presented in Figure 6. Monomer conversion was monitored using FTIR spectroscopy on the inkjet-printed sample from Figure 4a and is depicted in Figure 6a. The spectrum was normalised to the carbonyl bond at 1700 cm^−1^. The peak located at 1637 cm^−1^ corresponded to the stretching mode of the vinyl group and showed full consumption after curing. Additionally, the water contact angle as an important surface characteristic was evaluated at 57.5° (Figure 6b) which is in the moderate hydrophilic region for polymeric materials for TE scaffolds [64].

The degradation profile is presented in Figure 6c. It has been well reported that ester moieties in polyesters are capable of undergoing hydrolytic degradation [65] with FDA approved polyvinyl alcohol as a degradation product making them suitable for medical implants [15,16,66]. Consequently, we conducted mass loss studies at pH 10. The results revealed a linear degradation rate, leading to an 80% reduction in initial mass within 74 days, followed by a significant drop to 20% of the initial mass after 94 days and full degradation after 106 days. Such degradation kinetics aligned with the typical behaviour of polyesters [67].

Photopolymers utilised for biomedical applications potentially release unreacted resin components into their vicinities. While previous research has reported low cytotoxicity of VEs using washing procedures between 24 to 48 h to precondition the test specimens, it is important to note that our study encountered challenges in reproducing the short washing procedures employed by other groups [16,25]. In our case, a more extensive washing process was required to achieve biocompatibility (75% ethanol for 4 × 24 h at 37 °C under constant agitation followed by 4 × 24 h in growth medium under the same conditions). The extended washing procedure might be attributed to the inhibitor, which ensures sufficient shelf and pot life.

Under physiological conditions, the eluted resin components showed no relevant influence on the viability of BMSCs within 24 h (Figure 6d). We detected a viability of 84% at the highest eluate concentration and according to ISO 10993-5 [68], a percentage of cell viability of more than 70% is considered noncytotoxic. In addition, cells were seeded directly on the scaffold and cultivated for 72 h and stained for living/dead cell status. BMSCs were able to adhere, showed living cells of typical stromal cell morphology and spread on the material (Figure 6e).

Mechanical properties are presented in Table 3 and Figure 6f–i. DMA measurements indicated a glass transition temperature (T_g_) of −24.4 °C and a storage modulus (E’) of 9.76 MPa at 37 °C. Tensile strength was measured at 1.19 MPa and compression strength was determined to be 4.2 MPa. The bulk material exhibited a relatively soft network, making it well-suited for the fabrication of soft TE scaffolds [69,70,71].

## 4. Conclusions and Outlook

Results of the study demonstrated the additive and lithographic processability of a thiol-ene photocurable resin, across different scales with remarkable fidelity, employing material jetting, vat photopolymerisation and nanoimprint lithography. The resin exhibited suitable properties with respect to viscosity, surface tension, absorption spectra and thermal stability to be employed in four distinct technologies, including 3D inkjet printing with PolyJet^TM^ technology and on a PixDro LP50, DLP and NIL. Our experiments confirmed the fabrication of bioinspired structures, covering six orders of magnitude, from porous lattices with different unit cells in the millimetre range to patterned surfaces such as pillars, grooves and moth eye structures in the micro/submicro range. In addition to fabrication technologies, the characterisation of bulk material properties revealed noncytotoxicity, degradability and mechanical properties suitable for soft bioinspired scaffolding structures. Nevertheless, some challenges still need to be addressed and a set of future research directions are therefore proposed. First, the material and processing technologies provide an interesting platform for the creation of tissue engineering scaffolds for in vitro studies with cells, cytokines, chemokines and growth factors. However, to achieve implantable scaffolds, additional verifications are needed. Tissue engineering scaffolds are class III medical devices according to the EU MDR 2017/745 [72], unless they incorporate cells, in which case they are considered advanced therapy medicinal products (EC Regulation 1394/2007) [73]. Accordingly, regarding class III scaffolds, long-term evaluation of the biocompatibility should be performed. Second, our desire for biomimetic developments derives into multiscale and multimaterial manufacturing requirements, which the different technologies employed in this study could address in an even more synergic fashion, if research efforts focus on the integration of different manufacturing technologies and processable materials into a single machine chassis. Third, looking beyond tissue engineering we enter the realms of biofabrication and engineered living materials, for which biomaterials and living entities (i.e., cells and bacteria) are processed together to create biohybrid constructs. A remarkable research direction is foreseen in this respect, linked to embedding cells or even microorganisms within the thiol-ene photopolymer and evaluating if the processing of the “living resin,” employing vat photopolymerisation, material jetting and nanoimprint lithography, leads to viable biohybrid constructs and allows the cells and microorganisms to survive. In conclusion, the current manuscript expands the potential applications of VEs and opens new avenues for the fabrication of multiscale bioinspired structures.

## Figures and Tables

**Table 1:** Mass fraction of the used monomers, stabiliser and initiator and summary of the liquid resin properties.

Formulation	Concentration (wt.%)	Viscosity at 1000 s^−1^ (mPa·s)			Static Surface Tension at 25 °C (mN/m)	Density at 25 °C (g/mL)
		25 °C	40 °C	60 °C		
DVA	44.12	21.82	12.51	6.95	34.34	1.15
PETMP	54.38					
Propyl gallate	0.50					
BAPO	1.00					

**Table 2:** Increase in viscosity after 30 days of storage. Viscosity was measured (n = 30) at 100 s^−1^ and measured at storage temperature.

Day	Viscosity		Viscosity	
	at 25 °C (mPa·s)	Increase in %	at 60 °C (mPa·s)	Increase in %
0	21.36	-	6.81	-
10	21.58	1.03	7.55	10.87
20	21.77	1.92	7.97	17.03
30	22.03	3.14	8.66	27.17

**Table 3:** Bulk mechanical properties obtained from DMA, tensile testing and compression testing. Tensile and compression at 25 °C and a rate of 0.1 mm/s.

E’ (MPa)	T_g_ ^1^ (°C)	Tensile Strength (MPa)	Yield Strain (%)	Youngs Modulus (MPa)	Compression Strength (MPa)	Compression Strain (%)	Compression Modulus (MPa)
37 °C							
9.76	−24.4	1.19	17	7.5	4.2	31	12.5

^1^ derived from max tan delta.

## Data Availability

Data are contained within the article.
